# Progress in trial registration in Latin America and the Caribbean, 2007–2013

**DOI:** 10.26633/RPSP.2017.31

**Published:** 2017-03-23

**Authors:** Pablo Rodríguez-Feria, Luis Gabriel Cuervo

**Affiliations:** 1 Office of Knowledge Management, Bioethics, and Research Pan American Health Organization (PAHO) / Regional Office of the World Health Organization (WHO) Washington, DC United States of America Office of Knowledge Management, Bioethics, and Research, Pan American Health Organization (PAHO) / Regional Office of the World Health Organization (WHO), Washington, DC, United States of America

**Keywords:** Clinical trials as topic, health policy, research, ethics, policy, Pan American Health Organization, Caribbean region, Central America, South America, North America, Ensayos clínicos como asunto, política de salud, investigación, ética, políticas, Organización Panamericana de la Salud, Región del Caribe, América Central, América del Sur, América del Norte, Ensaios clínicos como assunto, política de saúde, pesquisa, ética, políticas, Organização Pan-Americana da Saúde, Região do Caribe, América Central, América do Sul, América do Norte

## Abstract

This descriptive study identifies trends in clinical trial registration in the World Health Organization International Clinical Trial Registry Platform (ICTRP) for Latin America and the Caribbean (LAC), from 2007–2013, and provides adjusted estimates for registration rates by population and publications (2007–2011). Trends and data are presented by subregion and language in interactive graphs, including annual registration rates by population (2007–2011) and publications (LILACS and MEDLINE) listed in SCIENTI Network (Science and Technology Indicators). Of the 11 945 clinical trials involving LAC countries, 8 282 were in South America, with Brazil leading at 4 070 (49%); 2 421 in North and Central America, with Mexico leading at 1 886 (78%); and 1 242 in the Caribbean, with Puerto Rico leading at 857 (69%). After adjusting by population and publication rates Chile, Panama, Argentina, and Peru led registration rates per 1 million inhabitants. Variations in the number of trials per year are quite substantial.

Clinical trial registration increased in a steady yet inconsistent way. The implementation of the Policy on Research for Health has been followed by an increase in countries that require registration and have established clinical trial registries. However, there is room for improvement in adherence throughout LAC. Trial registration is offered gratis by Brazilian, Cuban, Peruvian, and United States registries, among others.

## RESUMEN

En este estudio descriptivo se establecen las tendencias en cuanto al registro de ensayos clínicos en la Plataforma de Registros Internacionales de Ensayos Clínicos (ICTRP) de la Organización Mundial de la Salud (OMS) en América Latina y el Caribe en el período 2007-2013, y se incluyen cálculos ajustados de las tasas de registro por población y por publicaciones (2007-2011). Las tendencias y los datos se presentan por subregiones e idiomas en gráficos interactivos, y además se incluyen las tasas anuales de registro por población (2007-2011) y publicaciones (LILACS y MEDLINE) que figuran en la Red ScienTi (indicadores de ciencia y tecnología). De los 11 945 ensayos clínicos realizados en países de América Latina y el Caribe, 8 282 tuvieron lugar en América del Sur, en donde Brasil lleva la delantera con 4 070 (49 %); 2 421 se realizaron en América del Norte y Centroamérica, donde México se ubica en primer lugar con 1 886 (78 %), y 1 242 se realizaron en el Caribe, donde la mayoría de los ensayos fueron en Puerto Rico, con un número de 857 (69 %). Después de ajustar las tasas por población y publicaciones, Chile, Panamá, Argentina y Perú tuvieron las tasas más altas de registro por 1 millón de habitantes. Hubo amplias variaciones en el número de ensayos clínicos por año.

El registro de ensayos clínicos aumentó de manera constante, aunque no uniforme. La ejecución de la Política de investigación para la salud de la Organización Panamericana de la Salud (OPS) llevó a un aumento del número de países que han establecido registros de ensayos clínicos y que requieren que se realice este registro. Sin embargo, podría mejorarse la observancia de esa política en América Latina y el Caribe en su totalidad. El registro de ensayos es gratuito en Brasil, Cuba, Estados Unidos y Perú.

## RESUMO

Estudo descritivo que identifica as tendências no registro de ensaios clínicos na Plataforma Internacional de Registro de Ensaios Clínicos (ICTRP) da Organização Mundial da Saúde (OMS) para América Latina e Caribe de 2007 a 2013, e apresenta estimativas ajustadas dos índices de registro por população e publicação (2007–2011). As tendências e os dados são apresentados por sub-região e idioma em gráficos interativos, incluindo os índices anuais de registro por população (2007–2011) e publicação (LILACS e MEDLINE) listada na SCIENTI Network (Science and Technology Indicators). Dos 11.945 ensaios clínicos realizados nos países da América Latina e Caribe, 8.282 foram conduzidos na América do Sul, na sua maioria (4.070) no Brasil (49%), 2.421 nas Américas Central e do Norte, na maior parte (1.886) no México (78%), e 1.242 no Caribe, em maior número (857) em Porto Rico (69%). Após o ajuste por população e publicação, Chile, Panamá, Argentina e Peru apresentaram os maiores índices de registro por 1 milhão de habitantes. A variação no número anual de estudos é bastante considerável.

Verificou-se um crescimento estável, porém inconsistente, no registro de ensaios clínicos. Com a implementação da Política para pesquisa em saúde, aumentou o número de países em que o registro de ensaios clínicos é exigido e que implantaram registros próprios. No entanto, é possível melhorar ainda mais a adesão na América Latina e Caribe visto que este registro é gratuito no Brasil, Cuba, Peru e nos Estados Unidos, entre outros.

Research for health should inform sound investments in the health sector (1). Trial registration is used as a strategy for promoting transparency and trust in research and is promoted by PAHO and WHO policies.

In 2015, WHO reiterated its position on requiring clinical trial registration (2, 3). Also, the BMJ called for research transparency for non-regulated interventions (e.g. diets, exercise programs, surgical procedures) to understand the harms and benefits of those interventions (4).

In 2009, PAHO/WHO Member States approved the “Policy on Research for Health” (the Policy; 5). The Policy called for better governance and transparency in research, and the strengthening of research systems that inform policies for health, prevention, and health care. This first-of-its-kind, Regional policy addressed nearly 30 years-worth of calls to improve research transparency using trial registration. It also called for a better use of the International Clinical Trial Registry Platform (ICTRP), the WHO meta-registry of clinical trials that organizes standardized information on 20 variables from approved data providers (6). Additionally, the “Strategy on Research for Health,” adopted at the World Health Assembly in 2010, mandated clinical trial registration (7).

The Policy focuses on strengthening the building blocks of “research for health systems,” and trial registration affects several of its key objectives. For example, trial registration contributes “to foster best practices and enhanced standards for research” and to promote transparency; it also improves access to information. It is essential to “maintaining public trust, confidence, and participation in research.” Hence, it calls for Member States to “fulfill research registration and enable inventories and registries of research comparable and integrated with the ICTRP primary registries, and to adopt standard identifiers and data-set collections that contribute to international registration efforts and international ethics and publications standards” (1). Furthermore, clinical trial registration facilitates knowledge translation, the identification of competent human resources for research, and the development of partnerships.

Clinical trial registration had a boon during the 2004 Ministerial Forum on Health Research in Mexico leading to the subsequent development of the ICTRP. The initial assessment of registration in Latin America and the Caribbean (8) found a 17-fold, yet inconsistent, increase in clinical trial registration during first quinquennium of ICTRP (9).

**FIGURE 1.**
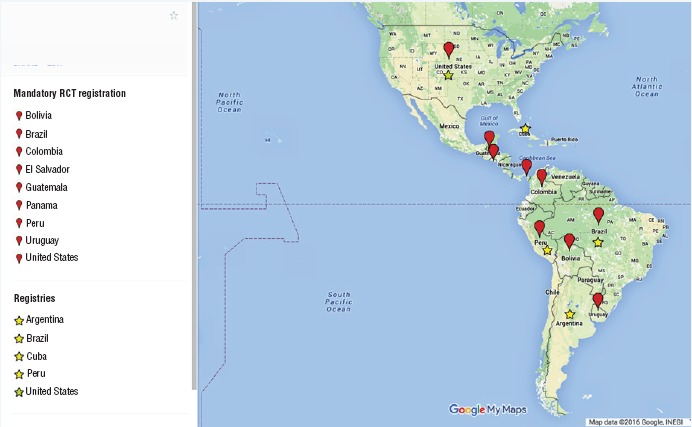
Countries in the Americas with mandatory Registration of Clinical Trials (RCT) and those developing registries, 2015

***Source:*** Adapted with the permission from the EQUATOR Annual Lecture on 30 September 2015. Available from: www.equator-network.org/2015/10/02/annual-lecture-2015-luis-gabriel-cuervo/

Clinical trial registration has been promoted through different entry points (Figure 1), such as national legislation requiring registration in publicly accessible databases, or through national clinical trial registries (10). In some countries, agencies request proof of trial registration to fund research. Some require registration to publish research reports, approve a project, or allow for ethical approval (9–11). The scientific community and the World Medical Association have been among the many stakeholders and ethics experts advocating for such transparency and governance measures (12, 13), together with publishers and scientific journals that have made registration a requirement, even if compliance needs to be enhanced (9).

The implementation of the Policy is being assessed, as well as adherence to clinical trial registration since much has changed since ICTRP was set up. For example, the Ministerial Forum on Research for Health called for “…standards, regulations, and best practices for fair, accountable, and transparent research processes; guaranteeing quality and safety of patient care; registration and results reporting of clinical trials; open and equitable accessing to research data, tools, and information”(14).

Trial registration has remained a key strategy for transparency and dissemination of findings (15) and for informing initiatives, such as the Global Research for Health Observatory requested by the World Health Assembly in 2013 (16). Other stakeholders complement these efforts by improving transparency and public trust in research for health. For example, +AllTrials (17, 18), the REWARD Initiative (19–24), Equator Network (25), the Lancet, the BMJ, other leading journals (26, 27) and The Cochrane Collaboration all advocate for research registration.

The present study assessed registration trends in ICTRP, pulling together data from 15 primary registries and 3 other data providers (28). *Primary registries* meet WHO standards capturing data on the 20 items of the required minimum data set; other data providers meet some of these standards (10) and may not have a national or regional remit, or the support of the government, nor be open to registration by all.

The study objectives were: to describe the trends among clinical trials registered in ICTRP and being carried out in Latin America and the Caribbean (LAC) in 2007–2013; to provide adjusted registration rates by population and publication for 2007–2011; to deliver helpful recommendations for improving research governance; and to support transparency in research, increasing its value and reducing waste.

## MATERIALS AND METHODS

This was a descriptive analysis of clinical trials from LAC countries registered in ICTRP, following the protocol developed by one of the authors (PRF) as part of a research internship with PAHO/WHO.

### Inclusion and exclusion criteria

The data was comprised of clinical trials registered in the ICTRP and being conducted in LAC (29). The advanced search function of ICTRP was used to identify these trials. The searches were run on 11–22 August 2014. The study used the WHO definition of clinical trial: “any research study that prospectively assigns human participants or groups of humans to one or more health-related interventions to evaluate the effects on health outcomes (30).”

Countries not listed in ICTRP would have no trials registered. The countries and overseas territories excluded were not listed in the ICTRP because they had no registered trials.2 Duplicate studies identified as such by ICTRP filters or algorithms were excluded using the identification number and date of registration.

### Data extraction and analysis

Registered trials were identified by year, whether conducted in a single country or as part of a multi-country, multicentric study. The data were stratified by geographic distribution within LAC, and by language, as follows:

### Geographic distribution

- North/Central America: Costa Rica, El Salvador, Guatemala, Honduras, Mexico, Nicaragua, and Panama.
- Caribbean: Bahamas, Barbados, Belize, Cuba, Dominican Republic, Guadeloupe, Guyana, Haiti, Jamaica, Martinique, Puerto Rico, Saint Kitts and Nevis, Suriname, and Trinidad and Tobago.
- South America: Argentina, Bolivia, Brazil, Chile, Colombia, Ecuador, Paraguay, Peru, Uruguay, and Venezuela.

### Language

- Spanish: Argentina, Bolivia, Chile, Colombia, Costa Rica, Cuba, Dominican Republic, Ecuador, El Salvador, Guatemala, Honduras, Nicaragua, Mexico, Panama, Paraguay, Peru, Puerto Rico, Uruguay, and Venezuela.
- Portuguese: Brazil.
- Other: Bahamas, Barbados, Belize, Guadeloupe, Guyana, Haiti, Jamaica, Martinique, Saint Kitts and Nevis, Suriname, and Trinidad and Tobago.

The dominant language in each population was considered, e.g., Spanish in Puerto Rico.

Google Motion Chart™ (Google Inc., Mountain View, California, United States) was used to illustrate registration trends by geographic distribution and language. The top three countries per geographic area are highlighted, along with countries showing notable variations or low registration.

## RESULTS

The search identified 11 945 clinical trials registered in January 2007–December 2013, with the following geographic distribution:

- North/Central America: 2 421 (20.3%)
- Caribbean: 1 242 (10.4%)
- South America: 8 282 (69.3%)

Population-and publication-adjusted rates are available from the interactive graphs in the Supplementary Materials, enabling readers to explore trends by country, language, and geographic area (Box 1).

### Geographic area

***North/Central America***. Mexico, Guatemala, and Panama led in this area with 1 886 (77.9%), 203 (8.4%), and 152 (6.3%) registered trials, respectively. Honduras with 28 (1.2%) and Nicaragua with 6 (0.2%) had the least (Table 1).

Mexico had 233 clinical trials registered in 2007, followed by a high of 332 in 2012 and 259 in 2013. The most growth occurred in 2009–2010, rising from 232 registered trials to 272.

Registered clinical trials in Guatemala increased steadily in 2007–2013, from a low of 18 in 2007 to a high of 38 in 2012.

Panama registered 12 clinical trials in 2007, increasing to 28 by 2013. Although 233% is an impressive increase, it may be far from accurately reflecting clinical trial development in this country; adherence is probably low.

In contrast, clinical trial registrations in Costa Rica plunged from 33 in 2007, to 2 in 2013. This decline occurred because in May 2010, the nation’s Constitutional Court imposed a moratorium on clinical trials while new regulations were implemented; the moratorium was lifted in 2014.

***Caribbean.*** Puerto Rico, Cuba, and the Dominican Republic led trial registration with 857 (69.0%), 218 (17.6%), and 91 (7.3%), respectively. Conversely, Suriname, Trinidad and Tobago, and Saint Kitts and Nevis had the lowest registration during these 7 years: 1 registration for Suriname and 0 from the other two. This most likely reflects poor compliance; research institutions in these countries conduct clinical trials on a regular basis (Table 1).

Puerto Rico led registration peaking in 2011 (151 trials), but decreased since then; only 74 trials were registered in 2013, a 49% reduction.

Cuba’s trends oscillated from 6 trials in 2007 to 56 trials in 2010, with the steepest increase in 2008–2009 (22 to 53 trials), concurring with the country’s establishment of the Cuban Public Registry of Clinical Trials Primary registry.

BOX 1. **Internet links for viewing the clinical trials registration trends and yearly rates, adjusted by population or publications, in Latin America and the Caribbean**

**Yearly rates for clinical trials registration**/population: https://docs.google.com/spreadsheets/d/1UKoUpR6SBIhlcQ476R9nZPwARSYPyY91VEfuh_0CWgc/pubchart?oid=686909007&format=interactive

**Yearly rates for clinical trials registration**/LILACS: https://docs.google.com/spreadsheets/d/1UKoUpR6SBIhlcQ476R9nZPwARSYPyY91VEfuh_0CWgc/pubchart?oid=1025092234&format=interactive

**Yearly rates for clinical trials registration**/MEDLINE: https://docs.google.com/spreadsheets/d/1UKoUpR6SBIhlcQ476R9nZPwARSYPyY91VEfuh_0CWgc/pubchart?oid=1423024517&format=interactive

**Trend of clinical trials registered in North America and Central America, by year:** https://docs.google.com/spreadsheets/d/1UKoUpR6SBIhlcQ476R9nZPwARSYPyY91VEfuh_0CWgc/edit#gid=734250587

**Trend of clinical trials registered in the Caribbean, by year**: https://docs.google.com/spreadsheets/d/1UKoUpR6SBIhlcQ476R9nZPwARSYPyY91VEfuh_0CWgc/pubchart?oid=1162517644&format=interactive

**Trend of clinical trials registered in South America, by year:** https://docs.google.com/spreadsheets/d/1UKoUpR6SBIhlcQ476R9nZPwARSYPyY91VEfuh_0CWgc/pubchart?oid=254243024&format=interactive

**Trend of clinical trials registered in other languages, by year:** https://docs.google.com/spreadsheets/d/1UKoUpR6SBIhlcQ476R9nZPwARSYPyY91VEfuh_0CWgc/pubchart?oid=770435638&format=interactive

**Trend of clinical trials registered in Spanish language, by year:** https://docs.google.com/spreadsheets/d/1UKoUpR6SBIhlcQ476R9nZPwARSYPyY91VEfuh_0CWgc/edit#gid=155086706

***Source:*** Prepared by the authors with data from http://apps.who.int/trialsearch/ and http://www.ricyt.org/indicadores

The Dominican Republic had wavering growth trends in clinical trial registrations that oscillated from 15 in 2008 to 8 in 2009, peaking at 19 in 2011.

***South America.*** Table 1 shows Brazil dominating in South America with 4 070 trials (49.1%). Brazil was followed by Argentina with 1 541 (18.6%) and Chile with 860 (10.4%). Uruguay, Bolivia, and Paraguay had the lowest registration numbers: Uruguay, 41 (0.5%); Bolivia, 22 (0.3%); Paraguay, 15 (0.2%).

**TABLE 1.** Registration of clinical trials in Latin America and the Caribbean, 2007–2013

Country	Year						
	2007	2008	2009	2010	2011	2012	2013
Argentina	192	215	162	202	258	294	218
Bahamas	1	0	3	1	1	2	0
Barbados	0	0	1	0	1	0	1
Belize	0	3	1	1	5	1	1
Bolivia	2	4	2	6	4	3	1
Brazil	290	504	550	636	754	741	595
Chile	116	125	107	104	140	143	125
Colombia	87	94	75	134	144	146	114
Costa Rica	33	34	17	10	8	4	2
Cuba	6	22	53	56	31	15	35
Dominican Republic	7	15	8	10	19	15	17
Ecuador	19	15	8	9	11	15	13
El Salvador	3	4	4	2	10	8	7
Guadeloupe	0	1	0	1	0	0	2
Guatemala	18	32	25	30	30	38	30
Guyana	0	0	0	1	0	1	0
Haiti	0	1	3	0	5	3	1
Honduras	3	4	4	3	5	2	7
Jamaica	1	1	5	4	6	5	2
Martinique	0	1	0	3	0	2	3
Mexico	233	252	232	272	306	332	259
Nicaragua	2	1	2	1	0	0	0
Panama	12	21	14	26	26	25	28
Paraguay	2	3	3	2	1	2	2
Peru	104	95	100	104	127	119	73
Puerto Rico	129	147	122	118	151	116	74
St. Kitts and Nevis	0	0	0	0	0	0	0
Suriname	0	0	0	0	0	0	1
Trinidad and Tobago	0	0	0	0	0	0	0
Uruguay	5	7	3	9	2	9	6
Venezuela	16	25	22	16	15	13	20

***Source:*** Prepared by the authors from the study data.

Brazil developed a national registry and doubled its registrations during the study period. In 2007, it registered 290 trials, peaking at 754 in 2011, and then 595 trials in 2013.

Argentina’s registration grew slightly from 192 in 2007 to 218 in 2013, with several fluctuations in 2008 (215), 2009 (162), and 2010 (202).

Chile also grew its registration numbers slightly, from 116 in 2007 to 125 trials in 2008, peaking at 143 in 2012. The lowest number of registrations was 104 in 2010.

### Language

***Spanish.*** In addition to the leading Spanish-speaking countries already listed, others showed significant growth, such as Colombia where registered trials increased from 75 in 2009 to 114–134 trials in the following years. Peru had a modest increase from 104 trials in 2007 to 119 in 2012, but in 2013 saw a decrease to 73.

Venezuela and Ecuador had similar trends: Venezuela went from 13 to 25 trials, with a peak in 2008. Ecuador went from 8 to 19 trials, with a peak of 19 in 2007.

***Portuguese.*** Brazil is the only Portuguese-speaking country considered, and was described above. In terms of absolute figures, Brazil dominates registrations in LAC.

***Other languages.*** This group had the lowest registration: 76 trials were registered for 11 countries during these 7 years: Jamaica, Haiti, and Belize led with 24 (31.6%), 13 (17.1%), and 12 (15.8%) trials, respectively. No trials were registered for Saint Kitts and Nevis nor for Trinidad and Tobago. Despite having prominent research institutions, Jamaica registered 1 trial in 2007, and peaked at 6 in 2011; it is the only country in this group with annual registration. Haiti registered 0 trials in 2007, and peaked at 5 in 2011. Belize also had 0 registered trials in 2007 and 5 in 2011 (Table 1).

### Yearly registration rates, by publication and population

Clinical trial registration rates per 100 publications were estimated using data in LILACS and MEDLINE per year, according to the Network on Science and Technology Indicators for 24 countries in LAC for 2007–2011 (31).

### Adjusted rates for registration, by population and year

Clinical trial registration rates per 1 million inhabitants were analyzed. Chile led registration rates, with a range of 6.1–8.1 trials / 1 million inhabitants; then Panama, with 3.6–7.4; Argentina, 4.0–6.4; and Peru, 3.3–4.3.

As mentioned, Costa Rica’s registration plunged following a moratorium on clinical trials. In 2007, Costa Rica was leading registration in LAC with 7.7 trials / 1 million inhabitants, but fell to 1.7 trials by 2011 (Table 2).

**TABLE 2.** Adjusted clinical trials registration by population, Latin America and the Caribbean, 2007–2011

Country	Year				
	2007	2008	2009	2010	2011
Argentina	4.9	5.4	4.0	5.0	6.4
Bolivia	0.2	0.4	0.2	0.6	0.4
Brazil	1.5	2.7	2.9	3.3	3.9
Chile	7.0	7.5	6.3	6.1	8.1
Colombia	2.0	2.1	1.7	2.9	3.1
Costa Rica	7.7	7.7	3.8	2.2	1.7
Cuba	0.5	2.0	4.7	5.0	2.8
Ecuador	1.4	1.1	0.6	0.6	0.7
El Salvador	0.5	0.7	0.6	0.3	1.7
Guatemala	1.4	2.3	1.8	2.1	2.0
Guyana	0.0	0.0	0.0	1.3	0.0
Haiti	0.0	0.1	0.3	0.0	0.5
Honduras	0.4	0.5	0.5	0.4	0.6
Jamaica	0.4	0.4	1.8	1.5	2.1
Mexico	2.2	2.4	2.2	2.4	2.7
Nicaragua	0.4	0.2	0.4	0.2	0.0
Panama	3.6	6.2	4.1	7.4	7.0
Paraguay	0.3	0.5	0.5	0.3	0.2
Peru	3.7	3.3	3.4	3.5	4.3
St. Kitts and Nevis	0.0	0.0	0.0	0.0	0.0
Suriname	0.0	0.0	0.0	0.0	0.0
Trinidad and Tobago	0.0	0.0	0.0	0.0	0.0
Uruguay	1.5	2.1	0.9	2.7	0.6
Venezuela	0.6	0.9	0.8	0.6	0.5

***Source:*** Prepared by the authors with population data from the Network on Science and Technology Indicators.

## DISCUSSION

Overall, LAC increased its absolute and relative registration during the study period. Clinical trial registration rates adjusted by publications also increased, but there is room for improvement since many eligible trials appear to be missing.

Costa Rica was an outlier, with high registration rates by population and a sharp decline following the moratorium of 2010 (32). In 2011, the country’s legislative assembly approved the *Ley Reguladora de Investigación Biomédica* (Act Regulating Biomedical Research) to regulate biomedical research with humans, and created the National Health Research Council to guarantee research quality, the protection of human research subjects, and their rights. The Government of Costa Rica has to implement this act by developing further legislation (29, 32). The moratorium had a lasting impact and the country will need time and effort to regain its leadership in trial development and registration. It is unclear whether the dip in Costa Rica reflects a loss of clinical trial volume, a loss in registration, or both.

Clinical trials registration fluctuated in Argentina. In documenting barriers to trials registration, various issues were found: researchers were unfamiliar with registration platforms, other than Clinicaltrials.gov; few investigators knew the registration process; and responsibilities, especially for multicentric studies sponsored by the industry, were diluted. In a nutshell, researches did not feel compelled to check the registration status of their trials (33).

Brazil increased clinical trial registration during the 7-year study period and launched a WHO-accredited national primary registry; the country invested in its national health research governance and is decidedly supporting registration (34, 35). However, it faces challenges in increasing adherence, such as improving the standards for data collection in fields subject to interpretation (e.g., free text fields), and streamlining the registration process to make it swift and competitive (36).

Additional national and regional WHO-accredited primary registries may be necessary in the Americas since registries serve specific governance and monitoring requirements. However, efficiencies may be found in regional registries, and perhaps these can be aligned with regional integration agreements, such as CARICOM, or the Central American Integration System. Some countries may have the volume of trials to justify investing in a sustainable national registry, while others may find efficiency and sustainability in supporting a regional registry within an integrated system. Two national registries have been accredited as Primary Registries for ICTRP (the Cuban Public Registry of Clinical Trials and Brazilian Clinical Trials Registry); Peru is expected to complete its accreditation soon, but there is still room for growth (28).

Africa offered a collaborative model solving many challenges for trials registration through the Pan African Clinical Trial Registry (PACTR) established in 2007. This registry brought efficiencies, with only marginal increments in cost, and increased coverage from a national to a continental registry. PACTR also offered innovative options for registration that address local needs with adaptations for poor Internet connections (e.g., manual, email, and postal mail registration). By 2010, PACTR had doubled registration, with South Africa leading the trend (37).

An analysis done in Egypt of ICTRP and PACTR showed that registration began in 1999 and peaked for ICTRP in 2012 with 686 trials, and for PACTR in 2013 with 56 (38). Egypt trailed South Africa in registration.

In LAC, the launch of national registries has been followed by increased registration. Similarly, when Choi and colleagues assessed registration in the Korean Clinical Research Information Service established in 2010, they found 1 323 trials registered in 2010–2014 and a growing trend (39). While in Australia, Lam and colleagues found that researchers preferred the Australian New Zealand Clinical Trials Registry in 2008 – 2012, over clinicaltrials.gov (3 379 versus 1 764); many had duplicate registrations (40). Researchers in India found 1 826 trials registered in 2007–2011 using Clinical Trial Registry-India, with a growth trend of 3.7%. They concluded that the gradual increase was associated with having their own registry (41).

Globally, and following the launch of ICTRP, registration has increased sevenfold: from 3 294 trials in 2004 to 23 384 in 2013; this reflects improved adherence as trials have not grown at the same rate. Europe peaked in 2005; North America in 2005 and 2008; and Asia has seen a gradual, but steady increase since 2005 (42). In the Americas, registration is man dated by the Policy, and several countries have made it a legal requirement. Adjusting ICTRP registration by population, Chile, Panama, and Argentina are leading countries; however, absolute figures are dominated by Brazil and Mexico. Costa Rica, Nicaragua, Puerto Rico, and Peru had decreasing registration trends. In 2016, Peru launched its Primary registry. It would be interesting to assess how this influences registration rates. Monitoring and assessment, as conducted in Argentina, helps to identify and address adherence issues.

Although LAC countries have been improving their registration trends, the growth is inconsistent, dominated by some countries and leaving substantial room for improvement by others. Improvement requires working with countries, regulatory agencies, research sponsors, funding agencies, publishers, and ethics review committees. This means addressing limitations in the governance systems, promoting adherence, and providing incentives to increase the value of research and reduce research waste. Therefore, additional legal and technological efforts are needed, and we recommend replicating this assessment in 3 years. Monitoring and collecting data will provide actionable data to guide progress in Latin America and the Caribbean.

ICTRP allows the public, research observatories, sponsors, consumers, and researchers to link research proposals with outputs. It enables systematic reviewers to include unpublished or ongoing research, a practice that is becoming an increasing feature in Cochrane Reviews. Research institutions can organize and give visibility to their clinical trials, identify research partners, peers, and sponsors. This is being done, for example, in a cancer research systems mapping under way (43). Tutorials are becoming available to facilitate registration (44). Still, identifying duplicate clinical trials is a challenge and a science, requiring complex algorithms, something ICTRP advisors and developers had already foreseen. Duplicates are frequently identified comparing variables, such as countries of recruitment, inclusion and exclusion criteria, health condition(s), and intervention(s). However, sometimes these change over time or from one registry to another.

This study provides a benchmark for progress made by implementing the mandate of the PAHO Policy on Research for Health. The assessment can be replicated, and readers can further explore the data using the interactive graphs accessible from Box 1. It also offers actionable data, and even if it is impossible to get an accurate count of clinical trials in each country, registration is a good alternative and a tool for better governance.

Growth in registration, without substantial growth in publications, suggests better adherence to registration. This study informs progress on this aspect of the Policy and the WHO Strategy on Research for Health (4, 11). This guidance and further analysis, such as the extent of characteristics of registered research reports on specific topics, enables a variety of various consumers to better determine the links between clinical trials and health research agendas. This helps to better understand if research sponsors and national agendas are aligned with national and regional priorities.

### Limitations

This analysis does not address variations in the quality of registration for the 20-item dataset within or between registries. ICTRP registrations can be influenced by retrospective registration (done after recruitment) or when a registry is launched, a law enforced, a requirement is set up (by publishers, for example), or when study start-dates bring inaccuracies. Clinical trials are a subset of all the studies published, and compliance can be complicated and demand many resources. Considering that the denominator is unknown, the figures are a limited indicator. Yet, trends provide actionable data and an idea of whether a plateau has been being reached.

### Conclusions and recommendations

Significant progress was made with research registration in PAHO Member States, but these efforts must expand and scale up to achieve consistent adherence throughout the Region of the Americas. Trial registration brings transparency in research for health and has gained momentum and credibility in countries, such as Cuba and Brazil, where leadership in implementing WHO-endorsed primary registries has produced the first Spanish and Portuguese interfaces, respectively, while submitting data in English to the ICTRP. These two registries are open to trial registration from other countries and are now being complemented by the Peruvian registry.

Efforts and resources should focus on enhancing adherence to clinical trial registration, by, among other things:

- Advancing legislation to require registration of all research, or at a minimum clinical trials, observational studies, and systematic reviews, and the sharing of information for relevant studies with international platforms such as ICTRP.
- Encouraging research sponsors and ethics committees to require registration prior to disbursing or allocating funds, or issuing ethics approvals.
- Admonishing institutions and research sponsors to require that trials funded with public monies show proof of registration, enhancing transparency of the use of public resources.
- Promoting the systematic search of ICTRP to identify eligible trials, and the replication of studies, such as this one, to monitor and evaluate trends, characterize research, and facilitate good research governance and stewardship.
- Enhance the use of ICTRP by research managers to showcase their research and research capacities.

Furthermore, publishers are instrumental to increasing adherence when requiring proof of registration, and when displaying registration identification numbers, prominently in publications. Additionally, regulatory agencies can require that applications for medicines and technologies be backed by registered trials. Governments can promote trial registration when collaboration results in efficiencies, as done in Africa with the Pan African Clinical Trial Registry.

We recommend the development of regional registries especially for countries with low volumes of research, where efficiencies and sustainability are key. All countries should promote legislation and regulation that encourages research registration and transparency. For countries with high volumes of research and the need for additional specific information, it may make sense to have a national registry, even though the key components of registration may be satisfied by using the existing registries.

There are pros and cons to having a regional versus several national registries, especially in terms of logistics, efficiencies, and governance. High volumes of trials may justify having national registries, while efficiencies may favor regional registries when volumes are low. Typically, a registry requires at least two fulltime skilled professionals, a cost that makes sense when the volume justifies it. National registries can capture additional information of relevance to a country, becoming a tool of the regulatory and stewardship processes. Regional registries can facilitate standardization (27).

Countries with low trial volumes may benefit from using an international registry, thereby investing fewer resources in the technological tool, and focusing more on legislation and other means of improving adherence. Registration should be expanded to cover observational studies.

Considering the recommendations and local situation, stakeholders can advance strategies to promote transparency in research and adherence to international standards and mandates that foster best practices and standards for research, seek efficiencies and enhanced impact and appropriation of research, strengthen research governance and promote research agendas, and build on public trust and engagement in research.

#### Acknowledgements.

The authors wish to thank Lain Chalmers and Trudo Lemmens for their thoughtful insights during the first drafts of the manuscript; and Julia Rodriguez Abreu, Rita Lechuga, and Maria Fernanda Merino for their assistance with the final drafts.

#### Disclaimer.

Authors hold sole responsibility for the views expressed in the manuscript, which may not necessarily reflect the opinion or policy of the *RPSP/PAJPH* and/or PAHO.

## References

[1] 1. Pan American Health Organization. Policy on research for health. Proceedings of the 49th Directing Council, 61st Session of the Regional Committee of the World Health Organization for the Americas. Washington, DC: PAHO; 2009. Available from: www.paho.org/hq/index.php?option=com_content&view=article&id=1414&Itemid=931&lang=en Accessed on 19 May 2015.

[2] 2. World Health Organization. Statement on public disclosure of clinical trial results. Geneva: WHO; 2015. Available from: www.who.int/ictrp/results/reporting/en/ Accessed on 15 May 2015.

[3] 3. Moorthy VS, Karam G, Vannice KS, Kieny MP. Rationale for WHO's new position calling for prompt reporting and public disclosure of interventional clinical trial results. PLoS Med. 2015;12(4):e1001819. 10.1371/journal.pmed.1001819 PMC4396122 25874642

[4] 4. Dal-Rè R, Bracken MB, Ioannidis JP. Call to improve transparency of trials of non-regulated interventions. BMJ. 2015;350:h1323. 10.1136/bmj.h1323 25820265

[5] 5. Pan American Health Organization. Policy on research for health. Proceedings of the 49th Directing Council, 61st Session of the Regional Committee of WHO for the Americas, Washington, DC, 2009. Available from: www2.paho.org/hq/dmdocuments/2009/CD49-10-e.pdf Accessed on 11 January 2017.

[6] 6. Simes RJ. Publication bias: the case for an international registry of clinical trials. J Clin Oncol. 1986; 4:1529-41. Available from: www.jameslindlibrary.org/illustrating/records/publication-bias-the-case-for-an-international-registry-of-clin/key_passages Accessed on 16 January 2014. 10.1200/JCO.1986.4.10.1529 3760920

[7] 7. World Health Organization. The WHO strategy on reseach for health. Available from: www.who.int/phi/WHO_Strategy_on_research_for_health.pdf Accessed on 8 September 2014.

[8] 8. Pan American Health Organization, World Health Organization. International clinical trials registry platform. Available from: www.paho.org/hq/index.php?option=com_content&view=article&id=1011&Itemid=3653&lang=en Accessed on 8 September 2014.

[9] 9. Reveiz L, Bonfill X, Glujovsky D, Pinzon CE, Asenjo-Lobos C, Cortes M, et al. Trial registration in Latin America and the Caribbean's: study of randomized trials published in 2010. J Clin Epidemiol. 2012;65(5):482-7. 10.1016/j.jclinepi.2011.09.003 22285461

[10] 10. Krleža-Jeriç K, Lemmens T, Reveiz L, Cuervo LG, Bero LA. Prospective registration and results disclosure of clinical trials in the Americas: a roadmap toward transparency. Rev Panam Salud Publica. 2011;30(1):87-96. 22159656

[11] 11. Pan American Health Organization, World Health Organization. Ethics Review Committee standard operating procedures for submitting research proposals. Geneva: WHO; 2009. Available from: www.paho.org/hq/index.php?option=com_content&view=article&id=3291%3A2010-ethics-review-committee-standard-operating-procedures-submitting-research-proposals&catid=2502%3Apublications&lang=es Accessed on 1 January 2017.

[12] 12. World Medical Association. Ethical principles for medical research involving human subjects, 2013. Available from: www.wma.net/en/30publications/10policies/b3/ Accessed on 8 September 2014.

[13] 13. Andrew E, Anis A, Chalmers T, Cho M, Clarke M, Felson D, et al. A proposal for structured reporting of randomized controlled trials. The Standards of Reporting Trials Group. JAMA. 1994;272(24):1926-31. Available from www.jameslindlibrary.org/illustrating/records/a-proposal-for-structured-reporting-of-randomized-controlled-trials/key_passages Accessed on 16 January 2015. 7990245

[14] 14. Bamako Call to Action on Research for Health: Strengthening research for health, development and equity. Global Ministerial Forum on Research for Health. Bamako, Mali, 17-19 November 2008. Available at www.who.int/rpc/news/BAMAKOCALLTOACTIONFinalNov24.pdf Accessed on 18 Jan 2017.

[15] 15. Lemmens T. Pharmaceutical knowledge governance: a human rights perspective. J Law Med Ethics. 2013;41(1):163-84. 10.1111/jlme.12012 23581664

[16] 16. World Health Organization. Follow up of the report of the Consultative Expert Working Group on Research and Development: financing and coordination. Proceedings of the 66th World Health Assembly. Resolution 66.22. Available from: http://apps.who.int/iris/bitstream/10665/150173/1/A66_R22-en.pdf?ua=1&ua=1 Accessed on 7 January 2017.

[17] 17. Chalmers I, Glasziou P, Godlee F. All trials must be registered and the results published. BMJ. 2013;346:f105. 10.1136/bmj.f105 23303893

[18] 18. Goldacre B. Are clinical trial data shared sufficiently today? BMJ. 2013;347:f1880. 10.1136/bmj.f1880 23838460

[19] 19. Macleod MR, Michie S, Roberts I, Dirnagl U, Chalmers I, Ioannidis JP, et al. Biomedical research: increasing value, reducing waste. Lancet. 2014;383(9912):101-4. 10.1016/S0140-6736(13)62329-6 24411643

[20] 20. Moher D, Glasziou P, Chalmers I, Nasser M, Bossuyt PM, Korevaar DA, et al. Increasing value and reducing waste in biomedical research: who's listening? Lancet. 2015; 387(10027):1573-86. 10.1016/S0140-6736(15)00307-4 26423180

[21] 21. Chalmers I, Bracken MB, Djulbegovic B, Garattini S, Grant J, Gülmezoglu AM, et al. How to increase value and reduce waste when research priorities are set. Lancet. 2014;383(9912):156-65. 10.1016/S0140-6736(13)62229-1 24411644

[22] 22. Ioannidis JP, Greenland S, Hlatky MA, Khoury MJ, Macleod MR, Moher D, et al. Increasing value and reducing waste in research design, conduct, and analysis. Lancet. 2014;383(9912):166-75. 10.1016/S0140-6736(13)62227-8 PMC4697939 24411645

[23] 23. Al-Shahi Salman R, Beller E, Kagan J, Hemminki E, Phillips RS, Savulescu J, et al. Increasing value and reducing waste in biomedical research regulation and management. Lancet. 2014;383(9912):176-85. 10.1016/S0140-6736(13)62297-7 PMC3952153 24411646

[24] 24. Chan AW, Song F, Vickers A, Jefferson T, Dickersin K, Gøtzsche PC, et al. Increasing value and reducing waste: addressing inaccessible research. Lancet. 2014;383(9913): 257-66 10.1016/S0140-6736(13)62296-5 PMC4533904 24411650

[25] 25. Simera I, Moher D, Hirst A, Hoey J, Schulz KF, Altman DG. Transparent and accurate reporting increases reliability, utility, and impact of your research: reporting guidelines and the EQUATOR Network. BMC Med. 2010;8:24. 10.1186/1741-7015-8-24 PMC2874506 20420659

[26] 26. The Lancet. Research: increasing value, reducing waste. Available from: www.thelancet.com/series/research Accessed on 1 April 2015.

[27] 27. Laine C, Horton R, DeAngelis CD, Drazen JM, Frizelle FA, Godlee F, et al. Clinical trial registration. BMJ. 2007;334(7605):1177-8. 10.1136/bmj.39233.510810.80 PMC1889969 17548363

[28] 28. World Health Organization. The WHO Registry Network. Available from: www.who.int/ictrp/network/en/ Accessed on 10 December 2015.

[29] 29. Government of Costa Rica. Ley reguladora de investigación biomédica La Gaceta digital 2014. Available from: www.gaceta.go.cr/pub/2014/04/25/COMP_25_04_2014.pdf Accessed on 10 September 2014.

[30] 30. World Health OrganizationThe International Clinical Trials Registry Platform. Available from: www.who.int/ictrp/en/ Accessed on 11 January 2017

[31] 31. The Network for Science and Technology Indicators Ibero-American and Inter-American. Available from: www.ricyt.org/index.php?option=com_content&view=article&id=150&Itemid=20 Accessed on 11 January 2017.

[32] 32. Government of Costa Rica. Rinden dictamen afirmativo a la ley general de investigación en seres humanos. La Gaceta digital 2011. Available from: www.gaceta.go.cr/pub/2011/03/15/COMP_15_03_2011.pdf Accessed on 18 January 2017.

[33] 33. White L, Ortiz Z, Cuervo LG, Reveiz L. Clinical trial regulation in Argentina: overview and analysis of regulatory framework, use of existing tools, and researchers' perspectives to identify potential barriers. Rev Panam Salud Publica. 2011;30(5):445–52. 10.1590/s1020-49892011001100007 22262271

[34] 34. Villanueva EC, Abreu DR, Cuervo LG, Becerra-Posada F, Reveiz L, IJsselmuiden C. HR-Web Americas: a tool to facilitate better research governance in Latin America and the Caribbean. Cad. Saúde Pública [Internet]. 2012;28(10):2003. 10.1590/s0102-311x2012001000018 23090179

[35] 35. Alger J, Becerra-Posada F, Kennedy A, Martinelli E, Cuervo LG. National health research systems in Latin America: a 14-country review. Rev Panam Salud Publica. 2009;26(5):447–57. Available from: www.scielosp.org/scielo.php?script=sci_arttext&pid=S1020-49892009001100010&lng=en Accessed on 1 February 2001. 20107697

[36] 36. Freitas CG, Pesavento TF, Pedrosa MR, Riera R, Torloni MR. Practical and conceptual issues of clinical trial registration for Brazilian researchers. Sao Paulo Med J. 2015;134(1)28–33. 10.1590/1516-3180.2014.00441803 PMC10496585 26313113

[37] 37. Abrams AL. One of a kind--the Pan African Clinical Trials Registry, a regional registry for Africa. Pan Afr Med J. 2011;9:42. 10.4314/pamj.v9i1.71221 PMC3215564 22355440

[38] 38. Zeeneldin AA, Taha FM. The Egyptian clinical trials' registry profile: Analysis of three trial registries. J Adv Res. 2016;7(1):37–45. 10.1016/j.jare.2015.01.003 PMC4703417 26843968

[39] 39. Choi EK, Kim MJ, Lim NK, Park HY. Review of the registration in the Clinical Research Information Service. J Korean Med Sci. 2016;31(1):1–8. 10.3346/jkms.2016.31.1.1 PMC4712566 26770030

[40] 40. Lam J, Lord SJ, Hunter KE, Simes RJ, Vu T, Askie LM. Australian clinical trial activity and burden of disease: an analysis of registered trials in National Health Priority Areas. Med J Aust. 2015;203(2): 97–101. 10.5694/mja14.00598 26175250

[41] 41. Selvarajan S, George M, Kumar SS, Dkhar SA. Clinical trials in India: Where do we stand globally? Perspect Clin Res. 2013;4(3):160–4. 10.4103/2229-3485.115373 PMC3757579 24010056

[42] 42. Viergever RF, Li K. Trends in global clinical trial registration: an analysis of numbers of registered clinical trials in different parts of the world from 2004 to 2013. BMJ Open. 2015;5(9):e008932. 10.1136/bmjopen-2015-008932 PMC4593134 26408831

[43] 43. Lee B, Cuervo LG, Rodríguez-Feria P, Luciani S. Analysis of registered cancer clinical trials in Latin America and the Caribbean, 2007–2013. Rev Panam Salud Publica. 2016;39(2):115–21. 27754521

[44] 44. Pan American Health Organization, World Health Organization. How to register a Clinical Trial following international standards. Available from: https://www.youtube.com/playlist?list=PL15AE0B4DE63F9CCB Accessed on 11 January 2017.

